# Using poster presentation to assess large classes: a case study of a first-year undergraduate module at a South African university

**DOI:** 10.1186/s12909-019-1863-9

**Published:** 2019-11-21

**Authors:** Andrew Ross, Thembelihle Dlungwane, Jacqueline Van Wyk

**Affiliations:** 1 Discipline of Family Medicine, School of Nursing and Public Health, College of Health Sciences, University of KwaZulu-Natal, Durban, 4041 South Africa; 2 Discipline of Public Health Medicine, School of Nursing and Public Health, College of Health Sciences, University of KwaZulu-Natal, Durban, 4041 South Africa; 3 Academic leader for Research, School of Clinical Medicine, College of Health Sciences, University of KwaZulu-Natal, Durban, 4041 South Africa

**Keywords:** Education, Deep learning, Assessment, Posters, Feedback

## Abstract

**Background:**

The massification of higher education is often associated with poor student engagement, poor development of their critical thinking, inadequate feedback and poor student throughput. These factors necessitate the need to devise novel, innovative methods to teach, assess and provide feedback to learners to counter the restrictions imposed due to the large class learning environments. This study was conducted to ascertain the perceptions of 1st year medical students and staff at the Nelson Mandela School of Medicine regarding the value of poster presentations as a strategy to enhance learning, assessment and feedback.

**Methods:**

This was an exploratory observational, descriptive cross-sectional, case study. Data was collected through separate student and staff questionnaires that required participant responses on a five-point Likert scale. The data was extracted into Excel spreadsheets for quantitative analysis.

**Results:**

Two-hundred- and-thirty (92%) student questionnaires were returned (*N* = 250). Most students indicated that the design and presentation of the poster had helped them to select important material (92%), understand and describe disadvantage (86%) and to make a difference in the community (92%). The students agreed that the poster assessment was an efficient (81%) and fair method (75%) that provided opportunities for meaningful feedback. Ten staff members responded to the questionnaire. Most staff members (90%) indicated that the poster presentation had allowed students to demonstrate their engagement in a meaningful and appropriate way around issues of disadvantage and HIV and agreed that the poster presentations allowed for immediate and effective feedback.

**Conclusion:**

Students’ interactions in the tasks promoted active engagement with others and course material; the development of higher order thinking and skills which added to students’ accounts of transformative learning experiences. They could describe and illustrate the difference that they had made in their chosen community. The poster presentations allowed for quick and efficient marking, immediate feedback and an opportunity to validate the students’ participation.

Poster presentations offered an innovative way to encourage deep meaningful engagement and learning amongst peers and facilitators. Poster presentations should be more widely considered as an innovative way of encouraging deeper engagement and learning in a large class setting.

## Background

The South African National Development Plan has set targets that aim to increase the participation of students at Higher Educational Institutions (HEIs) by at least 70% by 2030. This translates into an estimated increased enrolment from 950,000 students in 2010 to approximately 1.62 million by 2030 [1]. Medical schools have not been exempted from the process [2–4] of massification of higher education due to the rapid increase in student enrolment [5]. At the Nelson R Mandela School of Medicine (NRMSM) of the University of KwaZulu-Natal (UKZN), the first year enrolment increased from 150 in 1990 to the current 250, while the members of staff who train on the programme have not increased proportionally [6].

The massification of higher education, compounded by an inadequate number of academic staff [4], often results in staff resorting to the use of didactic teaching strategies, when teaching students. This instructional method of teaching correlates poorly with the attainment of higher order learning [5, 7], as it usually leads to superficial learning that resembles an accumulation of factual information (Blooms taxonomy level 1) rather than deep engagement, insight, analysis and transformative learning. Mezirow (2003) describes transformative learning as learning that challenges fixed assumptions that people may have to make them more inclusive, open, reflective and emotionally able to change [8]. It is envisaged that transformative learning, as a desirable outcome of tertiary education, will develop students’ ability to discover new ways of being in the world [9], and result in students’ ability to demonstrate appropriate high order outcomes (levels 4, 5 6 of Bloom’s taxonomy) [10].

Didactic, lecture-based teaching as a characteristic of large classes is frequently associated with low student engagement with peers, staff and teaching material, and often manifests in low student motivation, superficial learning and poor student commitment [11]. This leads to a domino effect, where the students’ inadequate educational experiences and superficial engagement results in poor performance and higher numbers of students failing or dropping-out from their studies [12–15].

Assessment has been described as the single-most important determinant of learning, [16, 17] with students’ learning behaviour being greatly affected by the assessment strategy and the content to be assessed [18]. Identification of an assessment strategy that supports the ideals of deep learning is a major challenge in large classes, particularly in the context of staff shortages. As a result, many academics working at HEIs have resorted to the use of objective structured assessments, such as multiple- choice questions (MCQs), which mainly test students’ ability to recall, memorise and understand (levels 1 and 2 of Bloom’s taxonomy). There is thus a real and logistical challenge for those who must teach large numbers of students when selecting an assessment method, particularly when the skills to be assessed, would best be demonstrated by means of a student assignment.

In addition to the challenges associated with teaching and assessment as highlighted above, large class sizes makes effective feedback regarding student performance extremely difficult [19]. Compounding this challenge, Kulik and Kulik (1988), in their meta-analysis of 53 studies, highlighted the importance of feedback to be provided as soon as possible after the performance, if it is to have any impact on their learning [20].

Large classes may be associated with poor student engagement and lack of development of their critical thinking, inadequate feedback and poor student throughput [15]. In addition large classes can contribute to students’ lack of enthusiasm, confidence and motivation to learn [21]. However, this lack of engagement and critical thinking is not necessarily synonymous with large class teaching, but more with the presentation of the learning material, methods of assessment and provision of feedback [13]. It is therefore essential that lectures at HEIs develop novel and innovative methods of teaching, assessing and giving feedback to large groups of learners to counter the challenges posed by the large class learning environment [12, 22], especially for those educators working in resource constrained settings.

The Making a Difference (*MaD) group community service activity* that is offered at the NMSM presents an innovative opportunity for staff to facilitate significant community engagement and to find meaningful ways to assess large groups of students and provide immediate feedback. The MaD activity also offers first -year students an early, practical opportunity to make a difference in the lives of less fortunate others which may influence their worldview on health and sickness, advantage, disadvantage and privilege. This transformative learning opportunity shapes people and transformation as an outcome of learning at HEIs offers students deep and lasting change that results in a developmental shift [23].

*MaD* is a curriculated component of the ‘*Becoming a Professional’* (BaP) module required of 1st year medical students on a 6-year, integrated problem -based learning curriculum at UKZN. The students work in a group of four and spend a minimum of 16 h engaged in service and educational activities to the benefit of a disadvantaged community. Students are expected to self-select an accessible disadvantaged community for their *MaD* project. For the purpose of the MaD activity, a community was considered to be disadvantaged if it was socially or economically deprived. A final decision on the suitability of the community was made in conjunction with academic staff involved in running the MaD activity. Examples of such disadvantaged communities include old age homes, facilities for destitute indigent people, HIV and AIDS educational street activities, homes for abandoned babies, orphanages, children’s homes, places of safety, street children shelters and children living with disability. Each student group is expected to initiate and organise their own MaD activities with the host organisation. Over a 3-month period, the curriculum allows the group an afternoon per week to complete the activity. A faculty facilitator regularly meets with the group to assist with logistics and to provide support, facilitate debriefing and encourage student reflections on challenges encountered during the implementation of the educational activity. A previous publication has provided greater detail on the nature, purpose and types of facilities typically accessed by students when engaged in the *MaD group community service activity* [24]*.*

The nature of the service activities that groups undertake in MaD are planned in consultation with the host organisation according to the needs of the organisation. Each student keeps a diary and log of the time and activity and completes a set of structured reflective learning entries on an electronic journal. Students are required to analyse, synthesize and evaluate as they participated in the service and educational activities. Students also reflect on the constructs of being ‘disadvantaged’, the meaning of illness, and how to communicate HIV prevention messages in appropriate ways to those living in ‘disadvantaged’ communities. Each group of four students was required to design and present a poster to two faculty assessors and other groups of their 1st year student peers. This poster assessment contributes 25% towards the module mark and was the culmination of the students’ experiences and learning during their *MaD group community service activity*. The poster was expected to highlight the context, the activities undertaken during the 16 h of volunteer work and an evaluation of the impact and learning that had occurred during the *MaD group community service activity.* Each group was allocated 15 min for their poster presentation (10-min presentation and 5 min for questions). Sixty -three groups of four students each co-presented their work at the *‘Making a Difference Poster Day’,* with an average of four groups being assessed per hour by two faculty assessors and their peers using a standardized marking rubric. The group-poster presentation method was selected as an assessment tool due to the large number of students enrolled on the course and to ensure a fair and standardised way of assessing the work.

Although posters have been used for teaching and assessment in other settings [25], there is a scarcity of literature on the use of posters for teaching, assessment and to provide feedback to undergraduate medical students in the South African higher education environment. This study was therefore conducted to obtain the perceptions of students and staff regarding the value of the posters as a teaching, assessment and feedback strategy.

## Settings

Two hundred and fifty 1st year medical students were enrolled in the BAP module in 2018 and they identified 50 sites for their MaD projects (some sites had more than one group). Students visited the sites on four occasions between April 2018 and July 2018 to undertake the stipulated minimum period of 16 h of voluntary work. All the groups presented a poster detailing their experiences in October 2018. The cohort, their eight faculty supervisors and four assessors attended the poster presentations.

## Methods

This exploratory, observational, descriptive cross -sectional case study explored the perceptions of students and staff on the use of posters to manage the assessment and feedback to 250 first year students on their community project work at UKZN.

A 17- item student questionnaire (Additional file 1) was used at the end of the presentations to elicit student’s opinion on their engagement with the learning material, their learning during the *MaD: group community service activity*, the extent to which they preferred group over individual assessment, and their experiences of feedback and fairness during the assessment of their presentations. A 15-item staff questionnaire (Additional file 1) elicited the opinion of staff on the value of the posters as a learning, assessment and feedback strategy. A five -point Likert scale ranging from totally disagree to totally agree was used to assess both sets of responses, with totally disagree and disagree being consolidated into one response as was totally agree and agree responses. At the end of the student questionnaire, there were two open-ended questions which asked them to comment on whether they would have preferred an alternative method of assessment and to provide comments or suggestions on the poster presentation to the MaD team. Staff were asked whether they thought the MaD projects could have been assessed more efficiently using a different format. The questionnaire was developed by the authors and has been used for feedback and internal module evaluation.

The data was extracted into an Excel spreadsheet, and the responses to the open-ended questions reviewed in relation to the aim of the study. Ethical approval for the study was obtained from the Biomedical Research Ethics Committee of the institution (R201/04). All participants were informed of the nature and purpose of the study, assured of their anonymity and their right to withdraw at any stage. Signed informed consent was obtained from all participants prior to their participation in the study.

## Results

Two hundred and thirty (92%) completed questionnaires were returned of the 250 students who had been invited to participate. The students’ demographic breakdown indicated 220 (88%) Black; 30 (12%) Indian and four (1.6%) White students. Females comprised 56% of the class. Twelve members of staff worked in pairs to assess the 63 groups over a four- hour period, with two assessing each presentation. Ten (10/12) staff questionnaires were returned for analysis. Table 1 gives the students’ responses to the questions relating to learning, assessment and feedback.

**Table 1:** Student responses (*n* = 230)^a^

Statements	Disagree No. (%)	Neutral No. (%)	Agree No. (%)
Learning			
The poster preparation helped me to select important points for the presentation (*n* = 229)	1 (0.4%)	18 (8%)	211 (92%)
The poster presentation allowed our group to show why we considered this MaD community to be a disadvantaged group (*n* = 228)	3 (1.3%)	29 (13%)	196 (86%)
The poster presentation allowed me to demonstrate how I made a difference in this MaD community (*n* = 229)	3 (1.3%	15 (7%)	211 (92%)
The poster presentation allowed me to demonstrate my engagement in a meaningful and appropriate way around issues of HIV (*n* = 230)	5 (2.2%)	26 (11%)	199 (87%)
The poster presentation allowed me to demonstrate how I learnt from the participants at the MaD site (*n* = 227)	3 (1.3%	26 (11%)	198 (87%)
Posters are an effective way to show that I achieved the learning objectives of the MAD (*n* = 228)	7 (3.1%)	28 (12.2%)	193 (84.7%)
The poster preparation and presentation encouraged my interaction with my group (*n* = 226)	1 (0.4%)	14 (6%)	211 (93%)
Working in the group helped me learn more than what I would have learnt on my own (*n* = 226)	6 (2.7%)	19 (8%)	201 (89%)
Posters introduced a new way of sharing information for me (*n* = 228)	15 (6.6%)	38 (17%)	175 (77%)
I found the peer assessment to be beneficial to my learning (*n* = 228)	14 (6.1%)	35 (15%)	179 (79%)
This was my first time that I presented my work formally in the MBChB program	3 (1.3%)	8(4%)	213 (95%)
Assessment			
In my view, the poster presentation was an efficient form of assessment (228)	9 (3.9%)	35 (15%)	184 (81%)
In my view, poster presentations are a fair way to assess students	19 (8.4%)	38 (17%)	169 (75%)
Posters are an effective way for staff to validate our engagement with a disadvantaged community (*n* = 228)	10 (4.4%)	44 (19%)	174 (76%)
The peer assessment of posters helped clarify how marks are allocated (*n* = 228)	15 (6.6%)	44 (19%)	170 (74%)
The poster presentation made the assessment activity enjoyable (*n* = 228)	17 (7%)	38 (17%)	174 (76%)
Feedback			
The interactions with staff and peers provided opportunities for meaningful feedback (*n* = 165)	5 (3%)	16 (10%)	144 (87%)

^a^Not every question was answered by every student

In response to the question that asked about the use of an alternative method of assessment, 26 (14.2%) students indicated a preference to be assessed in an alternative way. Only 13 comments or suggestions were received of which six suggested the use of a Power Point presentation, two each for the inclusion of a site evaluation and a reflective summary respectively, one each suggesting feedback from the organisation, a video submission, or to be assessed by a test.

Table 2 gives the staff responses on the issues of learning, assessment and feedback.

**Table 2:** Staff responses (*n* = 10)

Statements	% Disagree	% Neutral	% Agree
Assessment of student learning			
The poster preparation enabled students to show why they considered this MaD Community as a disadvantaged group	0	0	100
The poster presentation allowed students to demonstrate how they had made a difference in this MAD Community	0	0	100
The poster presentation allowed students to demonstrate their engagement in a meaningful and appropriate way around issues of HIV	0	10	90
The poster presentation allowed students to demonstrate how they had learnt from the participants at the MAD site	0	10	90
The poster and presentation demonstrated that students interacted with their groups	0	10	90
Group work in the project and on the poster supported individual students to learn more than if they had worked alone	0	0	100
Assessment			
The poster presentations were a more efficient way to mark the work of 250 students than marking written assignments	0	0	100
In my opinion the posters are a fair method to assess the group	10	20	70
In my opinion the posters are a fair method to assess the individual student	0	0	100
The marking rubric was appropriate to assess the poster presentation	0	0	100
Feedback			
In my opinion, the posters are an effective way to provide feedback to all students	0	0	100
Interactions between staff and students allowed me to gain new insights into the scope of the community projects	0	10	90
Sufficient time was allocated for each poster presentation	0	10	90
Being part of the poster assessment team made the task of assessment more enjoyable	0	0	100
Engaging with the poster presentation with others from the same / different discipline offered a learning opportunity for me	0	10	90

Although one assessor indicated in the comments at the end of the questionnaire that he thought that an alternative method of assessment might be more efficient, he did not provide additional details.

## Discussion

The increased enrolments of students at HEIs have necessitated the development and use of appropriate teaching, assessment and feedback strategies to promote engagement and critical thinking to ensure that students achieve the stated learning outcomes of each module. Such activities should also be efficient in terms of their use of available time and human resources. The use of posters as a teaching, assessment and feedback strategy is relatively new in the MBChB programme at UKZN. Posters have however been used extensively with psychology and nursing students, and have been shown to promote collaboration and enjoyment in learning about research [17]. Such as reported in our study, researchers have similarly found that the educational use of posters increases students’ enjoyment of the learning process, helps create a positive learning environment, and assists with consolidation of their new knowledge. These factors greatly add to the confidence and motivation of the students to discuss research related topics [25].

A key challenge in teaching large classes is to promote deep learning, and to motivate students to actively engage with the learning material. The poster preparation and presentation required students to analyse their experiences, obtain feedback from participants, and evaluate the impact of the *MaD: group community service activity* on themselves and on those with whom they had worked. For students this form of assessment, promoted active engagement as they selected important experiences from their interaction with participants to demonstrate how they had understood ‘disadvantage’; had made a difference in the community; engaged with the community around issues of HIV and how they had learnt from the people at the site. Having to synthesise information for the poster thus promoted learning and active engagement as it challenged students to organise their learning experiences in a meaningful way to describe the benefits thereof. This promoted higher order thinking skills and deep learning [26].

Most staff members (90%) indicated that the poster presentation had allowed students to demonstrate their engagement in a meaningful and appropriate way around issues of “disadvantage” and HIV. Students also shared how they had learnt from their peers and others in the MAD community. The posters therefore allowed students to present to the assessors and their peers how their hands-on experiences at the service sites had culminated into new insights, transformational experiences and new learning.

All the students and staff members indicated that the poster presentation allowed students to demonstrate how they had made a difference in their selected MaD community. This is an important transformative outcome. First- year students needed to discover new insights about their ability to contribute meaningfully to a chosen community and that they did not have to wait until they were qualified as a medical practitioner to do so. Brooks et al. (2018) highlighted that students’ transformative learning experiences are enhanced when they feel that they are making a significant difference and are able to contribute to medical citizenship [27]. Coria et al. (2013) drew attention to the fact that assignments that ensure students confront real issues relating to social justice should be included in the medical school curriculum, as an increasing number of medical students want to find ways to decrease health disparities and are willing to work with underserved people [28].

Learning to collaborate and learn in teams is a key competency for health care professionals (HCP) to attain and an important core outcome competency, as specified by the Health Professions Council of South Africa (HPCSA) [29]. Designing assignments that promote students’ collaborative leaning in teams is therefore an important outcome on the MBChB curriculum that are assessed at various intervals during the 6-year programme. With increased polarisation of the South African society, there is a tendency among students to prefer to work in homogenous groups. However, due to the requirement for students to work in racially and culturally diverse groups on this task, they are encouraged to interact and learn about one another, and to work together in preparing and presenting their posters. Most students (89%; 201) indicated that working in groups had helped them to learn more than they would have learnt if they had worked alone. In addition, 93% (211) indicated that the poster preparation and presentation had encouraged interaction within the group. Researchers in other contexts have similarly recognised the value of posters to promote team work and learning from one another [25, 30]. Mellor identified group work as a way of supporting effective student learning [22], and highlighted benefits such as collaboration and socialisation [22] as they share their ideas, clarify differences, and develop conflict management skills that are develop during group interactions [12, 22].

Effective communication is another essential skill for HCPs to demonstrate [31]. This activity provided a safe and supportive setting for students to present their work on the MBChB (95%: 213). For most of the 1st year students (77%; 175) the poster presentation had introduced a new way to share information with others. Other researchers similarly reported the benefit of poster presentations as an excellent forum to develop communication and reflective thinking skills and opportunities for peer learning [25, 32]. Spiller highlighted the importance of clearly communicating the assessment requirements, methods and learning outcomes to students to ensure that learning is effective [16, 33]. When asked, 100% of the staff assessors indicated that the marking rubric provided consistency in the marking, and 75% considered the poster presentations to be a fair means of assessment to all students. However, only 60% of the staff indicated that it was fair to give the same mark to all members of the group, as it may not adequately discriminate between the contributions of each group member.

All the staff members indicated that marking the poster presentations in this multiple-reviewer context was quicker and more efficient than marking written assignments. The poster presentation ensured that all the marking was completed in a period of 4 h, allowed for immediate feedback, and for staff to validate student participation in the *MaD group community service activity*, which would not have been possible if they were marking a written assignment. Based on staff experiences in marking other written assignments in the same module, we estimated that it would have taken twice as long for two members of staff to mark each formal written group submission. In addition, a written assignment would have offered fewer opportunities to ask clarifying questions to validate the experience and therefore limit the feedback to the group.

All members of staff reported that probing questions posed to students about the meaning of disadvantage, how students had made a difference and had learnt from participants proved to be an effective way of validating the degree of the students’ involvement in the activity. However, only 76% of students indicated that the poster presentation validated their participation, which may have been because it was a group activity, and not all members may have participated equally. Plagiarism is a growing problem to counter on written assignments and a number of proactive and punitive mechanisms have been put in place at universities to ensure that students submit their own work and to combat plagiarism [34]. The poster presentations provided an easy and low-tech mechanism of validating student participation at their community site. More research is needed to find effective proactive ways to combat plagiarism and to ensure students participate and submit their own work.

Providing timely feedback is an important way to promote learning and engagement with the learning material [20, 35]. Feedback is said to develop self-awareness as students answer the following questions: ‘How am I doing?’ and ‘How can I get better?’ However, providing constructive feedback that is timely, meaningful, specific, effective, relevant to the current needs of the learner and gives suggestions to improve their learning [35] is difficult to provide in the large class context as well as when marking a large number of assignments, with students rarely getting adequate feedback. Most students (87%; 144) and staff (90%) agreed that the poster presentations allowed for immediate and effective feedback to groups about their presentation and the activities during the *MaD group community service activity.* It is important, even in large classes to find effective ways to give appropriate feedback to students to encourage their deep and meaningful engagement with the learning material. This area requires more research to find effective ways to provide feedback to large student groups enrolled at HEIs.

Although only 1.1% (26/ 230) of students wanted an alternative form of assessment, most students and staff were positive about the use of posters as a means of teaching, assessment and providing feedback. The alternatives suggested, such as power point presentations, would have taken more time to prepare and need additional logistical support. Site visits and direct feedback from the organisation have been built into student assessment in other countries, notably in the decentralized medical education in the Philippines [36]. Although this method of assessment is very effective in validating student experiences and evaluating the evidence of transformation in the communities, the site visit can be time consuming, costly and difficult to implement in a large class setting.

## Limitations

This case study was conducted with one cohort of *MaD* students at a medical school in a resource limited environment. Educators at other settings will have to compare our description of the setting and problem to assess the applicability of our results to their settings. However, the participation rate was very high, with the results being a good reflection of the student and staff perceptions at NRMSM of the use of posters for teaching, assessment and feedback.

## Conclusions and recommendations

The poster preparation and presentations encouraged student engagement with the learning objectives and collaboration with others. The posters assessed a range of generic and practical graduate competencies that require a higher level of learning as they needed to analyse and synthesize information for the presentation and allowed for feedback and validation of the student learning experiences. The presentations were viewed as an efficient and effective way of teaching, assessing and providing feedback to a large group of 1st year medical students. Poster presentations should be more widely considered as an innovative way to encourage deeper engagement and learning in a large class setting. Further local research and comparisons will help to identify practical and efficient methods to teach, assess and give feedback to encourage engagement, and deep learning among students attending HEIs in SA.

## Supplementary information

**Additional file 1.** Student questionnaire. Staff questionnaire.

## Data Availability

The datasets and materials used during this study are available from the corresponding author on reasonable request.

## Abbreviations

BaP: Becoming a Professional
HEIs: Higher Educational Institutions
*MaD*: Making a Difference
MCQs: Multiple- choice questions
NRMSM: Nelson R Mandela School of Medicine
UKZN: University of KwaZulu-Natal
