# Supplementary material for: Using poster presentation to assess large classes: a case study of a first-year undergraduate module at a South African university
Source: BMC Med Educ. 2019 Nov 21;19:432. doi: 10.1186/s12909-019-1863-9 (PMC6873480; doi:10.1186/s12909-019-1863-9)
Supplement: Supplementary file 1 — Additional file 1. Student questionnaire. Staff questionnaire. [file 12909_2019_1863_MOESM1_ESM.docx]

**Appendices**

**Student questionnaire**

Dear Student,

**Evaluation of the Making a Difference project**

Thank you for your participation in MAD project, presentation and assessment of the posters.   We value your feedback on the use of the poster preparation and presentation process to support your learning. Please use the **scale 1 - 5; where 1 = completely disagree and 5 is completely agree to** rate the following statements:

|  | **Agree Disagree** |
| --- | --- |
| 1. The poster preparation helped me to select important points for the presentation | **5 4 3 2 1** |
| 1. The poster presentation allowed our group to show why we considered this MAD community as a disadvantage group | **5 4 3 2 1** |
| 1. The poster presentation allowed me to demonstrate how I made a difference in this MAD community | **5 4 3 2 1** |
| 1. The poster presentation allowed me to demonstrate my engagement in a meaningful and appropriate way around issues of HIV | **5 4 3 2 1** |
| 1. The poster presentation allowed me to demonstrate how I learnt from the participants at the MAD site | **5 4 3 2 1** |
| 1. Posters are an effective way to show that I achieved the learning objectives of the MAD | **5 4 3 2 1** |
| 1. The poster preparation and presentation encouraged my interaction with my group | **5 4 3 2 1** |
| 1. Working in the group helped me learn more than what I would have learnt on my own | **5 4 3 2 1** |
| 1. This was my first time that I presented my work formally in the MBChB program | **5 4 3 2 1** |
| 1. In my view the poster presentation was an efficient form of assessment | **5 4 3 2 1** |
| 1. In my view the poster presentations are a fair way to assess students | **5 4 3 2 1** |
| 1. Posters are an effective way for academic staff to validate our engagement with a disadvantaged community | **5 4 3 2 1** |
| 1. Posters introduced a new way of sharing information for me | **5 4 3 2 1** |
| 1. The poster presentation made the assessment of this activity enjoyable | **5 4 3 2 1** |
| 1. I found the peer assessment to be beneficial to my learning | **5 4 3 2 1** |
| 1. The peer assessment of posters helped clarify how marks are allocated | **5 4 3 2 1** |
| 1. The interactions with staff and peers provided opportunities for meaningful feedback | **5 4 3 2 1** |

1. I would prefer an alternative method to assess the MAD project than a poster presentation **Yes / No**

If yes please suggest an alternative method of assessment: ____________________________________________

______________________________________________________________________________________________

**Any other comment/suggestions on the poster presentation for the MAD team**

**Thank you very much for your feedback. MAD Evaluation Team - Drs Knight, Van Wyk, Ross, Dlungwane**

**Staff questionnaire**

Dear staff member,

**Evaluation of the Making a Difference project**

Thank you for your participation in the Making a Difference project and the presentation and assessment of the posters.   We value your feedback on the use of the poster preparation and presentation process to support student learning. Please use the **scale 1 - 5; where 1 = completely disagree and 5 is completely agree to** rate the following statements:

|  | **Agree Disagree** |
| --- | --- |
| 1. The poster presentation enabled students to show why they considered this MAD community as a disadvantage group | **5 4 3 2 1** |
| 1. The poster presentation allowed students to demonstrate how they had made a difference in this MAD community | **5 4 3 2 1** |
| 1. The poster presentation allowed students to demonstrate their engagement in a meaningful and appropriate way around issues of HIV | **5 4 3 2 1** |
| 1. The poster presentation allowed students to demonstrate how they had learnt from the participants at the MAD site | **5 4 3 2 1** |
| 1. The poster preparation and presentation demonstrated that students interacted with their groups | **5 4 3 2 1** |
| 1. Group work in the project and on the poster supported individual students to learn more than if they had worked alone | **5 4 3 2 1** |
| 1. In my opinion the posters are a fair method to assess each student | **5 4 3 2 1** |
| 1. In my opinion the posters are a fair method to assess the group | **5 4 3 2 1** |
| 1. The poster presentations was an more efficient way to mark the work of 250 students than marking written assignment on this activity | **5 4 3 2 1** |
| 1. In my opinion posters are effective way to provide feedback to all students | **5 4 3 2 1** |
| 1. Sufficient time was allocated for each poster presentation | **5 4 3 2 1** |
| 1. The marking rubric was appropriate to assess the poster presentation | **5 4 3 2 1** |
| 1. Engaging in the assessment of the poster presentations with others from the same/different disciplines offered a learning opportunity for me | **5 4 3 2 1** |
| 1. Being part of the poster assessment team made the task of assessment enjoyable | **5 4 3 2 1** |
| 1. The interactions between staff and student allowed me to gain new insights about the scope of community projects | **5 4 3 2 1** |

1. Do you think that the Making a Difference project could be assessed more efficiently in a different format **Yes / NO**

If Yes please provide suggestion(s) below:

_________________________________________________________________________________________

**Any other comment/suggestions on the poster presentation for the MAD team**

**_________________________________________________________________________**

**MAD Evaluation Team - Drs Knight, Van Wyk, Ross, Dlungwane**
